# The anxiolytic effects of resistance exercise

**DOI:** 10.3389/fpsyg.2014.00753

**Published:** 2014-07-10

**Authors:** Justin C. Strickland, Mark A. Smith

**Affiliations:** Department of Psychology and Program in Neuroscience, Davidson College, DavidsonNC, USA

**Keywords:** anxiety, exercise, mental health, resistance, strength training

## Abstract

Numerous studies have revealed the beneficial effects of regular exercise across a variety of mental health measures. Although a great deal of attention has been paid to the role of aerobic exercise, less is known about the role of resistance exercise (i.e., strength training) in mental health outcomes. Resistance exercise includes a broad group of procedures that evoke repeated muscle action against resistances above those encountered in daily life. A growing body of literature has identified anxiolytic effects of resistance exercise in human populations after both single-bout sessions and long-term training. This research has shown that resistance training at a low-to-moderate intensity (<70% 1 repetition maximum) produces the most reliable and robust decreases in anxiety. Importantly, anxiolytic effects have been observed across a diverse range of populations and dependent measures. These findings provide support for the use of resistance exercise in the clinical management of anxiety.

Numerous studies have revealed a relationship between regular exercise and improvements in mental health, including increased cognition, mood, and general quality of life (Penedo and Dahn, 2005; Puetz et al., 2006). Although much of this research has examined the effects of aerobic exercise on mental health outcomes, resistance exercise (i.e., strength training) also produces many physiological and psychological benefits. In the only review of resistance exercise and mental health, increases in cognition, increases in self-esteem, and decreases in depression were noted across several randomized clinical trials (O’Connor et al., 2010). In addition to these effects, a growing body of evidence indicates that resistance exercise produces anxiolytic effects across a range of dependent measures, experimental procedures, and participant populations.

## RESISTANCE EXERCISE PROCEDURES

Resistance exercises include a variety of procedures that evoke repeated muscle action against resistances above those encountered in daily activities (Physical Activity Guidelines Advisory Committee, 2008). Resistance training usually requires the use of equipment, including elastic bands, free weights, or resistance machines, and it is performed in a series of sets that include a specific number of repetitions. A set of resistance exercise may involve combinations of concentric (i.e., shortening) or eccentric (i.e., lengthening) muscle movement with single- or multiple-joint action (American College of Sports Medicine, 2009). Resistance exercise intensity is usually measured as repetition maximums (RMs) with 1RM representing the maximal amount an individual can lift in a single repetition of a given exercise. Resistance training is more common in men than women, with 27% of males and 19% of females reporting regular resistance exercise (Schoenborn et al., 2013).

Numerous studies have documented the beneficial effects of resistance exercise on strength and performance-related outcomes, including increases in muscle mass, bone density, and endurance (e.g., see reviews by Crewther et al., 2006, 2011). This research also indicates that the benefits of resistance exercise extend beyond muscle and tissue growth and include alterations in neurobiological systems relevant to mental health and anxiety-related outcomes (e.g., cortisol and the HPA axis; Crewther et al., 2011). These studies are advancing our understanding of the role of resistance exercise in mental health by showing that resistance exercise produces robust alterations in the biological mediators of anxiety with potentially important implications for mental health outcomes.

## ROLE OF RESISTANCE EXERCISE IN ANXIETY

### MEASURING ANXIETY

As a construct, anxiety has been defined in a variety of ways (Endler and Kocovski, 2001). In the context of resistance exercise research, a distinction is made between anxiety as a state and anxiety as a trait. State anxiety is an acute emotional condition characterized by physiological arousal and accompanying feelings of tension and apprehension; in contrast, trait anxiety is the predisposition to respond in this state-conditional manner (Spielberger, 1966). The State-Trait Anxiety Inventory (STAI) is a 20-item measure of state and trait anxiety, with demonstrated reliability and validity (Spielberger, 1983). Other popular measures of anxiety include the Profile of Mood States (POMS), Hospital Anxiety and Depression Scale (HADS), and Symptom Checklist-90-R (SCL-90-R), all of which measure state rather than trait anxiety.

### ANXIOLYTIC EFFECTS OF SINGLE-BOUT RESISTANCE EXERCISE

The influence of a single session of resistance exercise on anxiety has been examined using both between-subjects and within-subjects designs, with most samples drawn from college populations and convenience groups (e.g., new enrollees in a weight-lifting course). Multiple studies have demonstrated the anxiolytic effects of single bouts of resistance exercise in human populations, and many of these studies have revealed a critical role for exercise intensity (see **Table 1**). Specifically, resistance exercise training that includes high intensities (i.e., >70% 1RM) is less likely to produce decreases in state anxiety than training with moderate or low intensities (i.e., 50–70% 1RM). Although significant decreases were not observed in many of the first studies to examine the effects of resistance exercise on state anxiety (Raglin et al., 1993; Koltyn et al., 1995; Garvin et al., 1997; Koltyn and Arbogast, 1998), all of those studies included resistance intensities at or above 70% 1RM. When exercise intensity has been reduced to 40–55% 1RM, acute decreases in state anxiety have been consistently reported (Bartholomew and Linder, 1998; Bibeau et al., 2010), and anxiolytic effects have been demonstrated at intensities as low as 10% 1RM (O’Connor and Cook, 1998).

**Table 1:** The effects of single-bout resistance exercise on anxiety measures.

Study	Sex	Age	*N*	Design (sampling)	Intensity (1RM)	Minutes	Measure	Anxiety outcome
Bartholomew and Linder (1998)	M/F	22	20	Within (recruit)	High (75–85%) vs low (40–50%)	20	STAI	High = increased and low = decreased
Bibeau et al. (2010)	M/F	20	18–24	Between (cluster)	High (80–85%) vs low (50–55%)	30	STAI	Decreased (greatest with low)
Focht (2002)	F	21	19	Within (cluster)	Self-selected vs fixed (75%)	30–45	STAI	Decreased
Focht and Koltyn (1999)	M/F	NA	28	Between (cluster)	50 vs 80%	30	STAI	Decreased (only at 50%)
Focht and Koltyn (2009)	M	21	21	Within (recruit)	75%	45	STAI	No change
Garvin et al. (1997)	M	22	15	Between (recruit)	70%	50	STAI	No change
Koltyn et al. (1995)	M/F	19	25	Between (cluster)	Self-selected (30–80% BW)	50	STAI	No change
Koltyn and Arbogast (1998)	M/F	23	13	Within (recruit)	75%	45	STAI	No change
O’Connor et al. (1993)	F	23	14	Within (cluster)	40% vs 60% vs 80% (10RM)	30	STAI	Decreased (only at 60%)
O’Connor and Cook (1998)	M/F	25	11	Within (cluster)	10%	30–45	STAI	Decreased
Parker et al. (2011)	M	23	18	Within (recruit)	70%	NA	POMS	No change
Passos et al. (2010)	M/F	42	12	Between (recruit)	50%	50	STAI	No change
Raglin et al. (1993)	M/F	20	26	Within (recruit)	70–80%	30	STAI	Increased

*RM, repetition maximum; M, male; F, female; cluster, from established group (e.g., weight training class); recruit, general recruitment; STAI, State-Trait Anxiety Inventory; POMS, Profile of Mood States; *N*, sample size in exercise group; age, mean age.*

Studies in which exercise intensities are directly compared also indicate a critical role for low-to-moderate resistance intensities. For instance, a single bout of resistance exercise at 45% 1RM produced decreases in state anxiety lasting until 120 min post-exercise, but the same effects were not observed at 30% 1RM or 60% 1RM (O’Connor et al., 1993). Similarly, exercise procedures performed at 50% 1RM but not 80% 1RM were shown to produce decreases in state anxiety, and this effect was not dependent on a previous history of resistance training (Focht and Koltyn, 1999). The duration of rest intervals between sets may also play an important role in the anxiolytic effects of resistance exercise, given that exercise performed at low intensities with long rests between sets (i.e., 50–55% 1RM and 90 s) produced robust decreases in state anxiety relative to high intensities with short rests (i.e., 80–85% 1RM and 30 s; Bibeau et al., 2010). In one study, anxiogenic effects were observed with resistance exercise in excess of 85% 1RM, but anxiolytic effects were observed at intensities of 50% 1RM (Bartholomew and Linder, 1998). It should be noted that a recent study conducted in middle-aged patients (30–55 years) with chronic primary insomnia failed to report a reduction in anxiety after a single bout of resistance exercise at 50% 1RM (Passos et al., 2010). It is unclear if the effects reported in that study were a consequence of the patients’ insomnia or their older age, given that few studies have examined acute bouts of resistance exercise in clinical populations or participants over 25 years of age. Regardless, most findings support the importance of low-to-moderate intensities when trying to maximize the anxiolytic effects of single-bouts of resistance exercise.

Gender may also play a role in the anxiolytic effects of resistance exercise, with females more sensitive to these effects than males. For instance, when given access to resistance exercise at either a self-selected intensity or a fixed intensity (75% 1RM), women exhibited robust decreases in state anxiety (Focht, 2002). These effects are particularly compelling considering single-bout anxiolytic effects have not been reliably demonstrated in men exercising at intensities greater than 70% 1RM (e.g., Garvin et al., 1997; Focht and Koltyn, 2009; Parker et al., 2011). Although some studies have failed to report sex differences (e.g., Bartholomew and Linder, 1998), the majority of findings suggest that females may be more sensitive to the anxiolytic effects of resistance exercise than males.

### ANXIOLYTIC EFFECTS OF LONG-TERM RESISTANCE TRAINING

In order to be effective in a clinical population, the acute anxiolytic effects of resistance exercise should persist during long-term resistance training. Many studies have examined the role of long-term resistance training in anxiety outcomes and several of these studies have been conducted in clinical populations (see **Table 2**). To date, long-term resistance training has been examined primarily with parallel-groups (i.e., between-subjects) designs that use simple randomization and traditional control groups (e.g., waiting list controls; Norvell and Belles, 1993; Jette et al., 1996; Herring et al., 2012). Additionally, several studies have included social interaction as a component of control conditions (e.g., attendance at fitness centers or social groups without the prescribed resistance training; Cassilhas et al., 2007, 2010) in order to help control for the influence of these potentially confounding factors.

**Table 2:** The effects of long-term resistance exercise on anxiety measures.

Study	Population	Sex	Age	*N*	Design (rand)	Intensity (1RM)	Weeks	Days	Minutes	Measure	Anxiety outcome
Agil et al. (2010)	Postmenopausal	F	53	18	Between (simple)	NI	8	3	NI	SCL-90	Decreased
Aidar et al. (2012)	Ischemic stroke	M/F	52	11	Between (simple)	Self-selected	12	3	45–60	STAI	Decreased
Bircan et al. (2008)	Fibromyalgia	F	46	13	Between (simple)	Progressive	8	3	40	HADS	No change (trend)
Cassilhas et al. (2007)	Older (healthy)	M/F	68	20	Between (simple)	50% vs 80%	24	3	60	POMS	Decreased (in 50%)
Cassilhas et al., 2010	Older (healthy)	M	68	20	Between (simple)	80%	24	3	60	STAI	Decreased
Courneya et al., 2007a,b	Breast cancer	F	49	73	Between (stratified)	60–70%	17	3	15–45	STAI	No change (trend)
Herring et al. (2012)	GAD	F	26	10	Between (blocked)	35–50% + 5% weekly	6	2	46	PSWQ	Decreased (with aerobic)
Jette et al. (1996)	Older (healthy)	M/F	72	42	Between (simple)	Progressive	12–15	2	30	POMS	Decreases (greatest in M)
Norvell and Belles (1993)	Law officers	M	33	14	Between (simple)	Progressive	16	3	20	SCL-90	Decreased
Sjogren et al. (2006)	Office workers	M/F	46	90	Within (cluster)	30%	15	5	6-8	VAS	No change
Tsutsumi et al. (1997)	Older (healthy)	M/F	69	14	Between (simple)	75–85% vs 55–65%	12	3	NI	POMSSTAI	Decreased (greatest 55–65)
Tsutsumi et al. (1998)	Older (healthy)	M/F	69	12	Between (simple)	75–85% vs 55–65%	12	3	NI	POMSSTAI	Decreased (greatest 55–65)

*RM, repetition max; M, male; F, female; NI, not indicated; GAD, Generalized Anxiety Disorder; STAI, State-Trait Anxiety Inventory; POMS, Profile of Mood States; PSWQ, Penn State Worry Questionnaire; HADS, Hospital Anxiety and Depression Scale; SCL-90, Symptom Checklist-90; VAS, Visual Analog Scale; Rand, randomization; *N*, sample size in exercise group; age, mean.*

Concordant with single-bout outcomes, the effects of resistance training on anxiety are moderated by exercise intensity, with the most robust decreases observed at low-to-moderate intensities. For instance, in a 24-week, community-based intervention, greater decreases in anxiety were observed in older participants (65–75 years) performing exercise at 50% 1RM relative to those exercising at 80% 1RM (Cassilhas et al., 2007). These same effects were observed after a 12-week intervention, with low-to-moderate intensity exercise (55–65% 1RM) producing greater decreases in anxiety than high intensities (75–85% 1RM; Tsutsumi et al., 1997, 1998). In contrast, a work-place intervention using light resistance training (30% 1RM) failed to reduce anxiety outcomes, suggesting that an intensity threshold might exist for the anxiolytic effects of resistance exercise (Sjogren et al., 2006). Together, these findings support the importance of low-to-moderate intensities in conferring anxiolytic effects after resistance training.

Several studies have examined the effects of resistance exercise on anxiety in older populations (>60 years), with generally positive outcomes. Whether conducted in the laboratory (Cassilhas et al., 2007, 2010), community (Tsutsumi et al., 1997, 1998), or home (Jette et al., 1996), these studies indicate that participation in regular resistance exercise produces decreases in measures of state and trait anxiety in older populations. No study has compared the anxiolytic effects of resistance training across different age groups, although decreases have been observed in both middle-aged (mean age = 32.8 years; Norvell and Belles, 1993) and post-menopausal (mean age = 52.6 years; Agil et al., 2010) populations. Together, these findings indicate that resistance training decreases anxiety across age groups, and may be an effective intervention for older populations suffering from anxiety-related concerns.

A limited number of studies have been conducted in clinical populations with either primary anxiety or anxiety related to another condition. Randomized clinical trials in patients with fibromyalgia (Bircan et al., 2008) and patients with breast cancer undergoing chemotherapy (Courneya et al., 2007a,b) have revealed small but non-significant anxiolytic effects of resistance training. Significant anxiolytic effects were observed after resistance training during stroke rehabilitation (Aidar et al., 2012), which is consistent with the large body of literature supporting the use of resistance exercise for stroke patients (see review by Brogardh and Lexell, 2012). In the only study of primary anxiety symptoms, resistance exercise produced small but non-significant decreases in worry-related symptoms during a 6-week program in females with generalized anxiety disorder (Herring et al., 2012). When resistance training was combined with aerobic exercise, which alone failed to decrease anxiety symptoms, robust decreases in anxiety were observed. This effect suggests that resistance exercise may enhance the effects of other modes of exercise, or conversely, other modes of exercise may enhance the effects of resistance training. Although more data are needed regarding the effects of resistance training in clinical populations, these preliminary findings provide support for the feasibility and efficacy of resistance exercise in the treatment of anxiety as a primary and secondary symptom.

## FUTURE DIRECTIONS

The studies reviewed reveal a putative role for resistance exercise in anxiety-related outcomes; however, more studies are needed examining these effects in clinical populations. With 22% of the population over 13 suffering from anxiety disorders in a given year, treating anxiety symptoms poses a great challenge for the mental health community (Kessler et al., 2012). Unfortunately, a paucity of research has examined the anxiolytic effects of single bouts of resistance exercise in groups other than young healthy adults, namely convenience samples drawn from college populations. Furthermore, studies of long-term resistance training have largely been conducted in healthy populations, with only one study examining the effects of resistance training in the treatment of primary anxiety disorders. Additional information regarding clinical populations would help guide the design and implementation of resistance exercise-based interventions.

Although resistance exercise has demonstrated anxiolytic effects, the mechanisms mediating these effects are less clear. Numerous studies have demonstrated alterations in hypothalamic pituitary adrenal (HPA) axis function related to anxiety-related disorders. Specifically, anxiety often occurs concurrently with unanticipated or extended activation of the stress response along the HPA axis, resulting in hypervigilance, fear, and sympathetic dysregulation (Chrousos, 2009). Consequently, anxiety disorders can be thought of as a disorder of the HPA axis in the form of hyperactivity (e.g., OCD, Panic Disorder, Generalized Anxiety Disorder; Chrousos, 2009) or hypoactivity (e.g., posttraumatic stress disorder; Meewisse et al., 2007). Through modulation of cortisol activity, resistance exercise may affect anxiety at the level of the HPA axis (Crewther et al., 2006, 2011). An understanding of an individual’s response to stress and the associated changes along the HPA axis could give insight into the mechanisms mediating anxiety-related outcomes; however, in the context of resistance exercise, no study has examined cortisol and anxiety responses concurrently, making direct correlations between these outcomes impossible. Such studies would greatly aid our understanding of the biological mediators of the anxiolytic effects of resistance exercise.

Little is known about other central nervous system changes induced by resistance exercise and how these alterations might affect measures of anxiety and general mental health. The neurobiological effects of aerobic exercise have been well characterized, and it is thought that many of the positive outcomes on cognition and mental health are mediated through exercise-induced changes in brain-derived neurotrophic factor (BDNF) and monoamine neurotransmitters (Southwick et al., 2005; Hillman et al., 2009; Gomez-Pinilla and Hillman, 2013). The limited data describing the influence of resistance exercise on central nervous system activity indicate that its effects differ from aerobic exercise. For example, insulin-like growth factor 1 (IGF-1) is known to regulate learning and memory through hippocampal plasticity (Aberg et al., 2006), and IGF-1 is increased by resistance exercise in animals (Cassilhas et al., 2012) and humans (e.g., Cassilhas et al., 2007, 2010). In contrast, recent evidence indicates that BDNF is increased after aerobic exercise but is not affected by resistance exercise in laboratory animals (Cassilhas et al., 2012). More information will be needed regarding the centrally mediated effects of resistance exercise in order to elucidate these putative biological mechanisms.

Under both single-bout and long-term training conditions, resistance exercise produces anxiolytic effects in a variety of populations. Future research will be necessary to translate these effects to a broader clinical environment; however, enough data exist to begin making recommendations for the design and implementation of resistance exercise-based treatments for anxiety disorders.

## AUTHOR CONTRIBUTIONS

Both authors contributed to the literature search, initial draft of the manuscript, and revisions of the work. Both authors gave approval for the final version of the manuscript for submission and agree to be accountable for all aspects of the work.

## Conflict of Interest Statement

The authors declare that the research was conducted in the absence of any commercial or financial relationships that could be construed as a potential conflict of interest.
